# *Drosophila melanogaster* as a function-based high-throughput screening model for antinephrolithiasis agents in kidney stone patients

**DOI:** 10.1242/dmm.035873

**Published:** 2018-11-16

**Authors:** Sohrab N. Ali, Thamara K. Dayarathna, Aymon N. Ali, Tijani Osumah, Mohamed Ahmed, Tyler T. Cooper, Nicholas E. Power, Dongxing Zhang, Dajung Kim, Rachel Kim, Andre St. Amant, Jinqiang Hou, Thomas Tailly, Jun Yang, Len Luyt, Paul A. Spagnuolo, Jeremy P. Burton, Hassan Razvi, Hon S. Leong

**Affiliations:** 1 Division of Urology, Department of Surgery, Schulich School of Medicine and Dentistry, Western University, London, ON N6A 4V2, Canada; 2 Translational Prostate Cancer Research Laboratory, Lawson Health Research Institute, London, ON N6A 4V2, Canada; 3 Department of Urology, Mayo Clinic, Rochester, MN 55905, USA; 4 Department of Mechanical and Materials Engineering, Western University, London, ON N6A 5B9, Canada; 5 Department of Chemistry, University of Santa Barbara, CA 93106, USA; 6 Department of Chemistry, Western University, London, ON N6A 3K7, Canada; 7 Faculty of Food Sciences, University of Guelph, Guelph, ON N1G 2W1, Canada

**Keywords:** *Drosophila melanogaster*, Nephrolithiasis, Calcium oxalate, Fluorescent bisphosphonate, Intravital imaging

## Abstract

Kidney stone disease involves the aggregation of stone-forming salts consequent to solute supersaturation in urine. The development of novel therapeutic agents for this predominantly metabolic and biochemical disorder have been hampered by the lack of a practical pre-clinical model amenable to drug screening. Here, *Drosophila melanogaster*, an emerging model for kidney stone disease research, was adapted as a high-throughput functional drug screening platform independent of the multifactorial nature of mammalian nephrolithiasis. Through functional screening, the therapeutic potential of a novel compound commonly known as arbutin that specifically binds to oxalate, a key component of kidney calculi, was identified. Through isothermal titration calorimetry, high-performance liquid chromatography and atomic force microscopy, arbutin was determined to interact with calcium and oxalate in both free and bound states, disrupting crystal lattice structure, growth and crystallization. When used to treat patient urine samples, arbutin significantly abrogated calculus formation *in vivo* and outperformed potassium citrate in low pH urine conditions, owing to its oxalate-centric mode of action. The discovery of this novel antilithogenic compound via *D. melanogaster*, independent of a mammalian model, brings greater recognition to this platform, for which metabolic features are primary outcomes, underscoring the power of *D. melanogaster* as a high-throughput drug screening platform in similar disorders. This is the first description of the use of *D. melanogaster* as the model system for a high-throughput chemical library screen.

This article has an associated First Person interview with the first authors of the paper.

## Abstract (precis)

**Summary:** Chemical library screens using *Drosophila melanogaster* as a model of nephrolithiasis can be performed in a high-throughput and efficient manner, leading to candidate drugs with clinical potential in kidney stone patients.

## INTRODUCTION

Nephrolithiasis (kidney stones) is a common urological disorder affecting ∼10% of the population in industrialized nations (López and Hoppe, 2010; Romero et al., 2010). In the United States, the prevalence of stone disease has increased from 5.2% (1994) to 8.4% (2012) (Scales et al., 2012). Globally, the incidence and prevalence of nephrolithiasis demonstrates a similar trend and contributes significantly to the development of chronic kidney disease (CKD) (El-Zoghby et al., 2012; Romero et al., 2010). The economic burden of nephrolithiasis is substantial, with annual spending reported at $5.3 billion in the United States alone (Kovshilovskaya et al., 2012). Despite sizeable nephrolithiasis-related costs and morbidity, with 5-year recurrence rates approaching 50% in affected individuals, a complete understanding of nephrolithiasis on the molecular level remains lacking (Katsuma et al., 2002; Lotan et al., 2004). The pathogenesis of calcium oxalate nephrolithiasis, the most common stone subtype (80%), is multifactorial (Finkielstein, 2006). Predisposing factors include metabolic disorders, such as hypercalcuria, hyperoxaluria or hypocitraturia, and environmental factors, such as diet. These etiological elements disturb the metastable biochemical homeostasis of urine, ultimately culminating in crystal deposition and stone formation (Pak, 1998).

Medical treatment prevention strategies for calcium oxalate nephrolithiasis, which have remained relatively stagnant since the mid-1980s, vary based on underlying etiology. Current evidence suggests that potassium citrate and thiazide diuretics are effective agents in the prevention of calcium oxalate stone formation in hypocitraturia and hypercalcuria states (Qaseem et al., 2014; Reilly et al., 2010). In contrast, in primary and idiopathic hyperoxaluria, proportionately important sources of calcium oxalate stone formation, evidence is sparse for a consistently effective medical treatment. Pyridoxine has been suggested in the past based on several small nonrandomized clinical trials (Balcke et al., 1983; Mitwalli et al., 1988; Rattan et al., 1994). Newer approaches, such as probiotic and oxalate decarboxylase treatment, have generated mixed results (Moe et al., 2011; Xu et al., 2013).

Progress has perhaps been limited owing to a lack of suitable pre-clinical models that reliably recapitulate the pathophysiology of this disorder. Several animal models have previously been established for the study of nephrolithiasis, including rat, mouse, porcine and canine models (Khan, 1997). Historically, the most prominent among these has been the rat model of nephrolithiasis. The rat model relies on dietary manipulation or intraperitoneal injection of lithogenic agents (ethylene glycol, ammonium chloride or vitamin D3) to induce calculus formation (Khan, 1997; Liu et al., 2007). The use of this model has generated variable results with inconsistent stone formation. In addition, the nephrotoxicity of the lithogenic agents has limited overall model utility (Khan and Glenton, 2010; Khan et al., 2006).

*Drosophila melanogaster* has recently emerged as a promising model of human nephrolithiasis (Miller et al., 2013). A rapid reproductive rate and a fully mapped and mostly understood genome, in tandem with experimental simplicity, make *D. melanogaster* a particularly powerful model. The renal system of *D. melanogaster* consists of two discrete components: nephrocytes and Malpighian tubules (MTs). As a unit these components display a remarkable degree of similarity in form and function to the human nephron. Nephrocytes form a collection of cells around the heart and esophagus that filter waste products from hemolymph in a fashion analogous to the human glomerulus, generating urine by active transport of water, ions and solutes into the MT lumen (Hirata et al., 2012; Miller et al., 2013; Weavers et al., 2009). Recent work using *D. melanogaster* has successfully produced calcium oxalate-based stones present within the MTs of *D. melanogaster*, and identified the role of oxalate co-transporters (SLC26A6) and the role of excess zinc in stone formation (Chi et al., 2015). Contents of the MTs are subsequently moved via peristalsis into the hindgut, where they are mixed with the fecal matter being produced in that organ. We discovered that the stones formed in the MTs are also present within fecal matter and could provide a noninvasive way of determining stone formation *in vivo* (Cognigni et al., 2011).

With this discovery, we developed novel imaging techniques for visualizing and quantifying calcium oxalate stone burden in the *Drosophila* model of calcium oxalate nephrolithiasis. This led to the development of a functional high-throughput screening platform, allowing us to screen chemical libraries to identify novel compounds that exhibit antilithogenic activity *in vivo* and are ingestible. Although this model does not fully recapitulate mammalian calculi formation processes, we successfully identified compounds that were highly effective at halting calculus formation. Hydroquinone β-D-glucopyranoside, commonly known as arbutin, is a glycoside with hydroxyquinone (HQ) moieties, most commonly isolated from the *Arctostaphylos uva-ursi* or Bearberry dwarf shrub (Pop et al., 2009). Arbutin was seen to bind both free calcium ions as well as oxalate, serving as a dual antagonist to calcium oxalate crystallization. This discovery indicated that other novel antilithogenic compounds can be discovered in a similar manner, owing to the primarily acellular development of kidney stones.

## RESULTS

### Bisphosphonate probes bind to calcium oxalate calculi formed by *Drosophila*

**Fig. 1.**
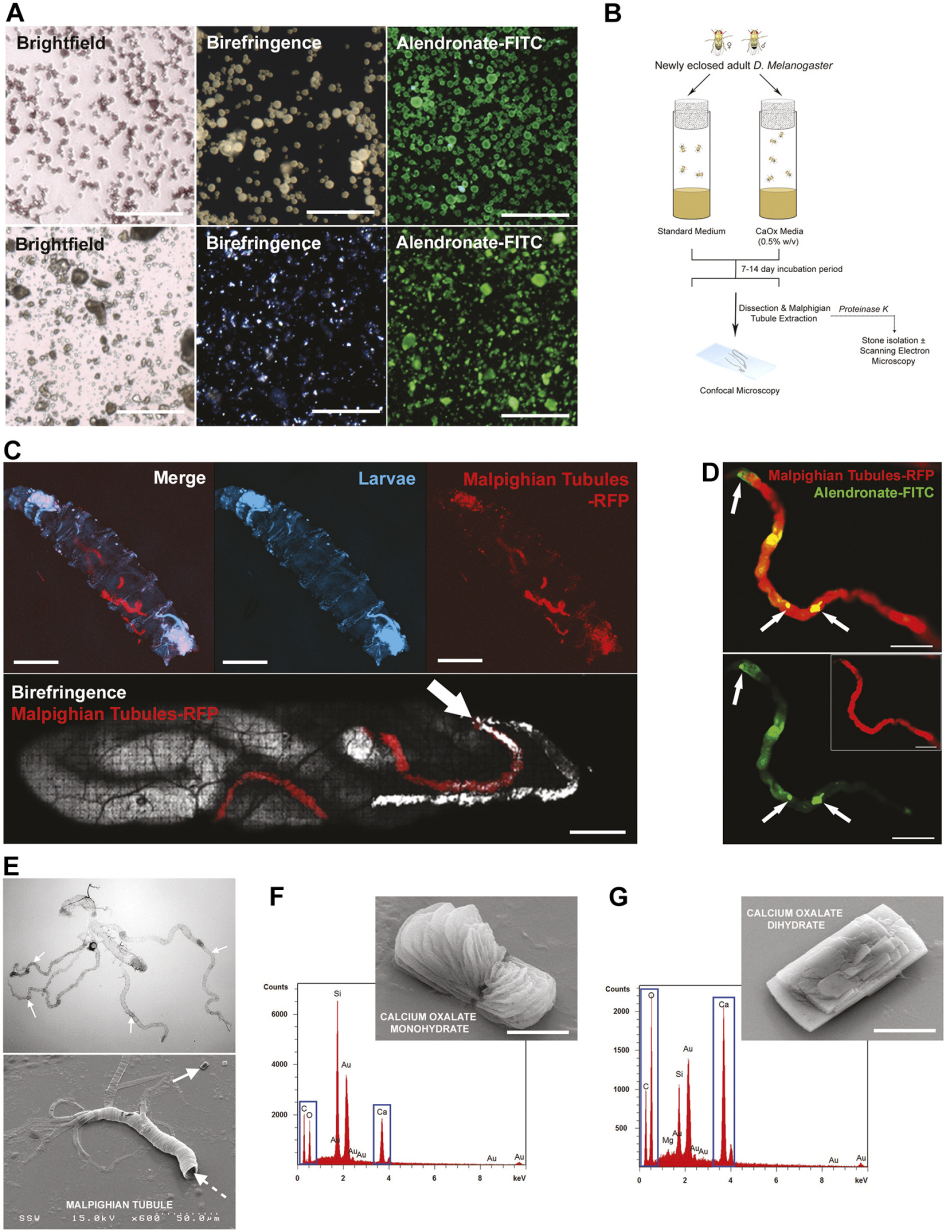
**Calcium oxalate calculi formation in *D. melanogaster*.** (A) Confocal and birefringence images of pulverized human calcium oxalate crystals (upper row) and synthetic hydroxyapatite particles (lower row) stained with alendronate-FITC probe. Scale bars: 1 mm. (B) Schematic of *D. melanogaster* model of calcium oxalate calculi formation within Malpighian tubules (MTs). (C) Intravital imaging of birefringence signal representing oxalate-based calculi within MTs of *D. melanogaster* larvae. Intravital imaging of RFP-expressing diet-induced calcium-oxalate-stone-carrying *D. melanogaster* larvae. Arrow indicates an RFP^+^ MT with birefringent signal (C, bottom). Scale bars: 1 mm. (D) Dissected MTs reveal the presence of alendronate-FITC-positive deposits (arrows) within RFP^+^ MTs, confirming the presence of oxalate-based calculi. Scale bars: 25 µm. (E) Dissected MTs as imaged by brightfield or scanning electron microscopy (SEM). Dashed arrow represents fly Malpighian tubule and gut complex. Solid arrow shows an MT calculus. (F) SEM/energy-dispersive X-ray (EDX) analysis of calcium oxalate monohydrate calculi extracted from MTs. Scale bar: 3 µm. (G) SEM/EDX analysis of calcium oxalate dehydrate calculi extracted from MTs. Scale bar: 3 µm.

We previously demonstrated that alendronate-fluorescein isothiocyanate (FITC), a fluorescently labeled bisphosphonate, binds to oxalate calculi via petrographic thin sections of calcium oxalate kidney stones and to nanocrystals in patient urine samples (Gavin et al., 2016). Pulverized calcium oxalate kidney stones were stained with alendronate-FITC and hydroxyapatite nanoparticles were used as a positive control, revealing a high binding affinity of the alendronate-FITC bisphosphonate probe to calcium oxalate calculi (Fig. 1A; Fig. S1A). Calcium oxalate calculi were formed in *Drosophila* by supplementing standard fly medium with varying amounts of sodium oxalate (0, 0.01, 0.05, 0.1, 0.5, 1.0% w/w) or ethylene glycol (0.1, 1, 2, 5% v/w). However, sodium oxalate produced the most consistent calculi formation in the MTs of *D. melanogaster* compared with polyethylene glycol (∼99% vs 93%, respectively). Newly eclosed *Drosophila* were added to fly tubes containing supplemented fly medium and incubated for 5-14 days (Fig. 1B). The GAL4-UAS system (Duffy, 2002), a potent tool for modifying gene expression, was utilized to develop transgenic fly lines to enhance imaging. Larvae were imaged for the presence of calculi within their MTs using *Drosophila* that express the fluorescent protein RFP in the MTs (UAS-RFP×URO-GAL4 cross) (Fig. 1C, top; Fig. S1B), with birefringence signal representing calculi within the MTs (Fig. 1C, bottom). MTs positive for birefringence signal were dissected and stained with alendronate-FITC, suggesting that formed calculi are calcium oxalate based (Fig. 1D; Fig. S2). Dissected MTs (Fig. 1E) and their resident calculi were submitted to energy-dispersive X-ray (EDX) spectroscopy, revealing the elemental composition of calculi to be calcium oxalate monohydrate (Fig. 1F) and dihydrate (Fig. 1G). This EDX spectroscopy analysis suggests that *Drosophila* produce calcium oxalate calculi *in vivo* that are elementally similar to those found in humans.

### *D. melanogaster* birefringence-positive fecal excreta as a high-throughput screening method for antilithogenic agents

**Fig. 2.**
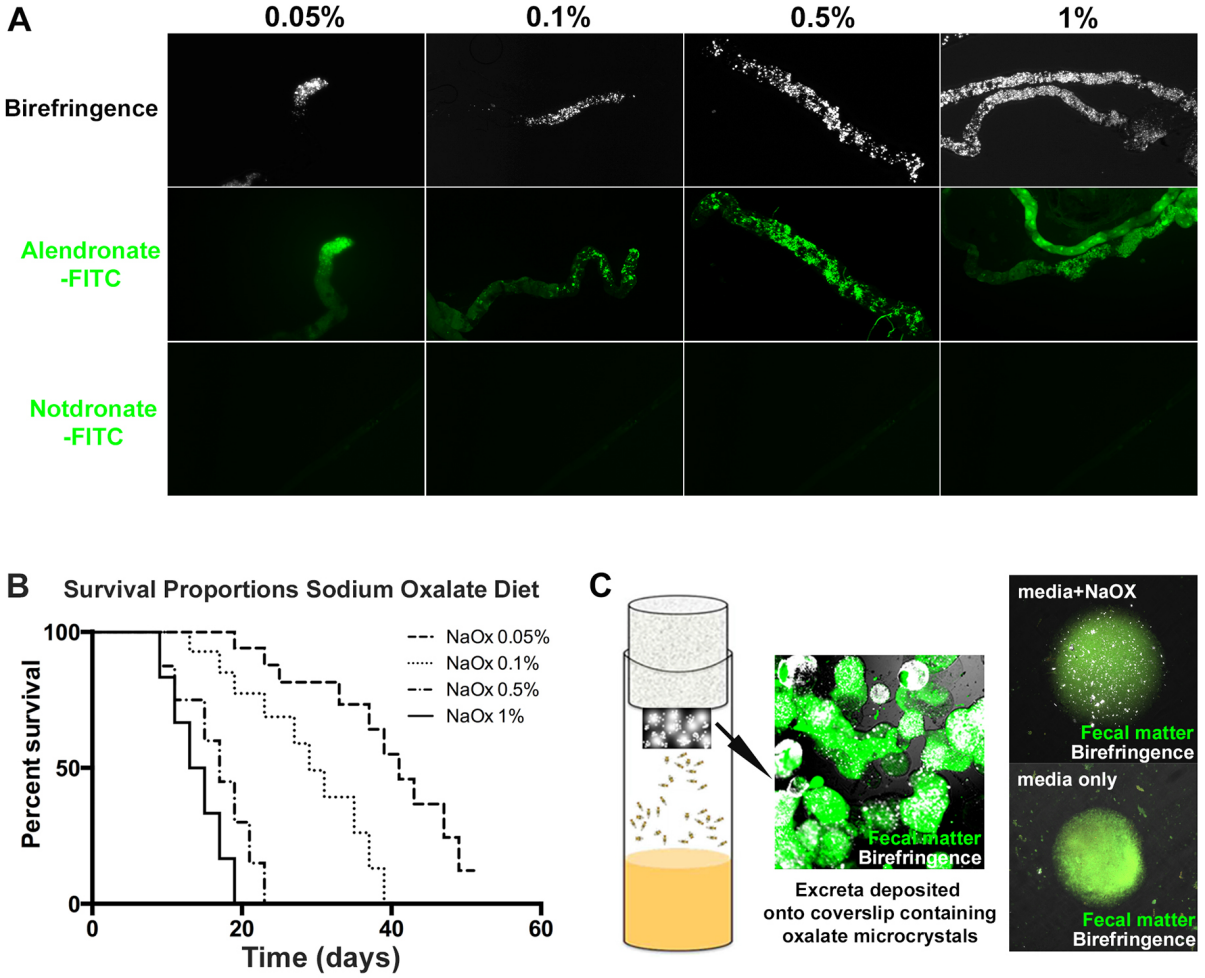
**Calculi present in fecal excreta of *Drosophila* as a drug discovery platform.** (A) Increasing amounts of sodium oxalate in fly medium induced kidney stone formation, present within MTs as evidenced by birefringence signal and alendronate-FITC signal. (B) *D. melanogaster* survival on sodium oxalate-treated fly medium over a 60-day period. (C) *D. melanogaster* deposit calculi-rich fecal excreta on the fly tube wall (green, autofluorescence of fecal matter) and coverslip attached to sponge lid. Flies grown in sodium oxalate-rich fly medium produce fecal excrement-containing birefringent bodies representing calculi (white, birefringence signal).

Oxalate-based calculi are found throughout the MTs in addition to the fly hindgut. Increased concentrations of sodium oxalate added to standard fly medium resulted in a proportional increase in stone burden within MTs. Birefringence signal (crystals) and alendronate-FITC staining (calcium oxalate) were utilized to quantitate stone/crystal burden (Fig. 2A). Increasing sodium oxalate concentration did, however, impact fly survival over time (Fig. 2B); therefore, a 0.5% w/v concentration of sodium oxalate was used in all subsequent experiments. A fecal excreta assay was developed to quantify changes in stone burden by inserting a coverslip in the sponge lid of fly tubes during incubation. *D. melanogaster* responded by passively depositing calculi-rich fecal excreta onto the coverslip (Fig. 2C, left and middle), which was then subjected to polarized light microscopy for birefringence signal representing crystals/calculi. Examination of a single fecal droplet revealed autofluorescence with highly birefringent mineral-like bodies within the fecal deposit. No such birefringent signal was observed in fecal deposits with a standard diet (Fig. 2C, right).

**Fig. 3.**
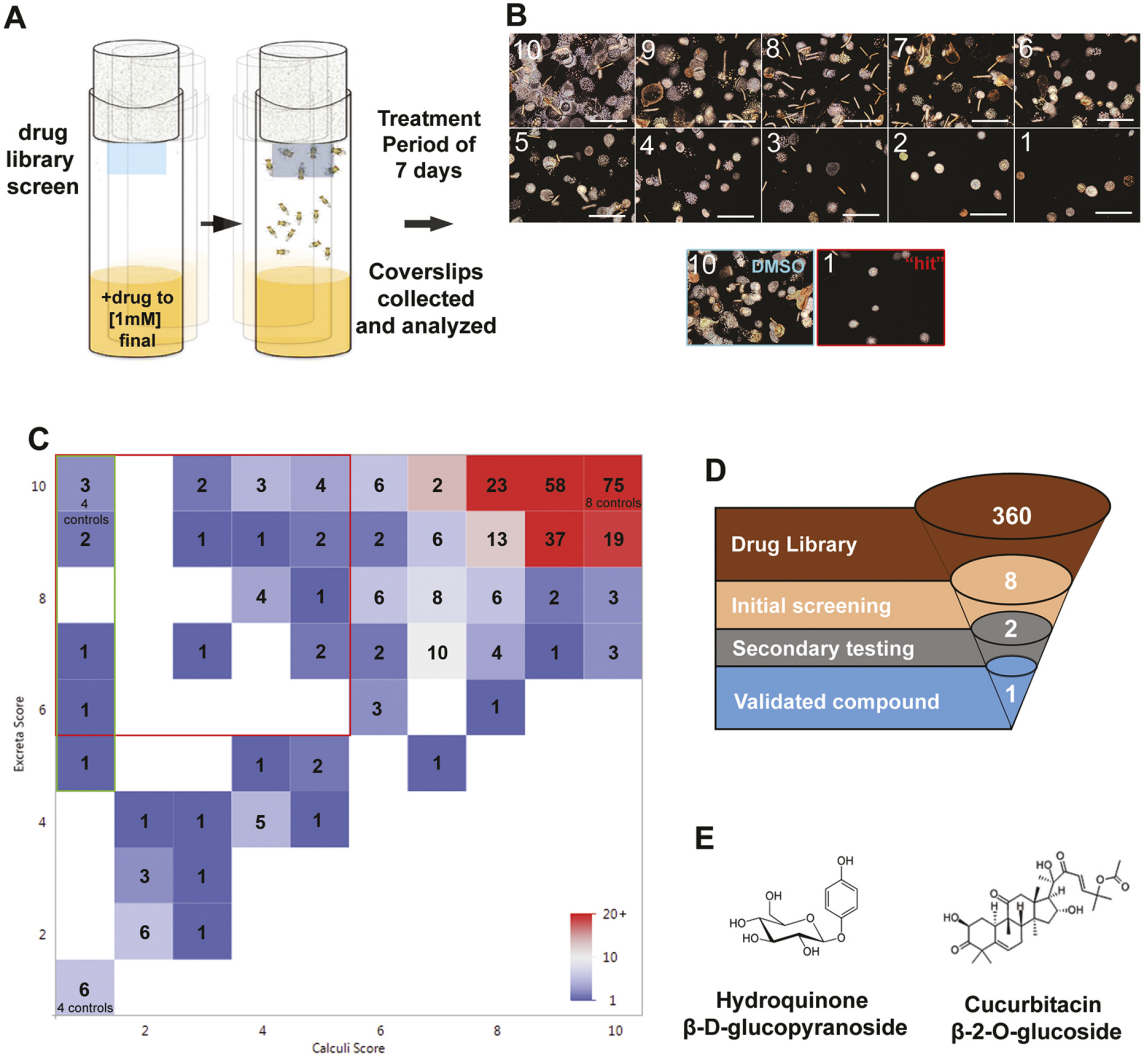
**Drug screening results using fecal excreta and calculi present within fecal excreta from *D. melanogaster* colonies.** (A) Schematic of *in vivo* drug library screens for antilithogenic compounds based on the calculi-fecal excreta coverslip assay. (B) Representative images of calculi or excreta density based on an arbitrary scale from 10 (highest/most dense) to 1 (lowest). A DMSO vehicle control with a score of 10 for calculi is shown. A representative ‘hit’ with a score of 1 for calculi is shown. Scale bars: 4 mm. (C) Drug screening results from a chemical library representing 360 naturally occurring compounds. ‘Hits’ were defined as coverslips that yielded a ‘1’ score for calculi deposition with no toxic impact on *D. melanogaster* viability (5-10 in terms of fecal excreta score, green gate). (D,E) A second focused library screen with the eight hits (D) yielded a final list of two active compounds (E). After tertiary analysis of the two active compounds, one was validated, arbutin.

A drug library screen (Lee et al., 2015; Rota et al., 2017; Spagnuolo et al., 2010) was performed, in which each drug was mixed into fly medium supplemented with 0.5% sodium oxalate to a final concentration of 20 µM drug (Fig. 3A). After 7 days of incubation and ingestion of drug candidates, the viability of flies was quantitated and all coverslips containing fecal excreta were analyzed for birefringence signal (Fig. 3B). Assessment of all coverslips was based on an arbitrary scale of 10 to 1 in terms of both fecal excreta (visual inspection of the entire coverslip) and calculi (via confocal microscopy of birefringence signal). Dimethyl sulfoxide (DMSO) controls were all 10 with regards to fecal excreta score and calculi score. A ‘hit’ was considered to be 10-5 in terms of fecal excreta scoring and 1 in terms of calculi score. Drug library screening (Fig. 3C, *n*=360 compounds), which included various negative and positive controls, yielded a minority of drug ‘hits’ that were toxic (1-5 in terms of fecal excreta score) and had no impact on calculi formation (*n*=28, 7.8%). The majority of candidate drugs did not impact *D. melanogaster* viability (5-10 in terms of fecal excreta score). Drug screening yielded a small number of drugs that decreased calculi score (1-5 in terms of calculi score) with minimal impact on *D. melanogaster* viability (5-10 in terms of fecal excreta score) (*n*=28; Fig. 3C, red gate, 7.8% of all drug candidates tested). Of these, eight were found to exhibit the greatest decrease in calculi formation (Fig. 3C, green gate). A second focused drug screen (*n*=8) was performed to confirm the antilithogenic effect *in vivo*; this second tier of analysis yielded two compound ‘hits’ that consistently exhibited antilithogenic activity *in vivo*; arbutin and cucurbitacin β-2-O-glucoside (Fig. 3E). Of these, arbutin was chosen as a proof of principle to validate the potential of *D. melanogaster* as a high-throughput model for drug screening.

### Arbutin is an effective antilithogenic agent

**Fig. 4.**
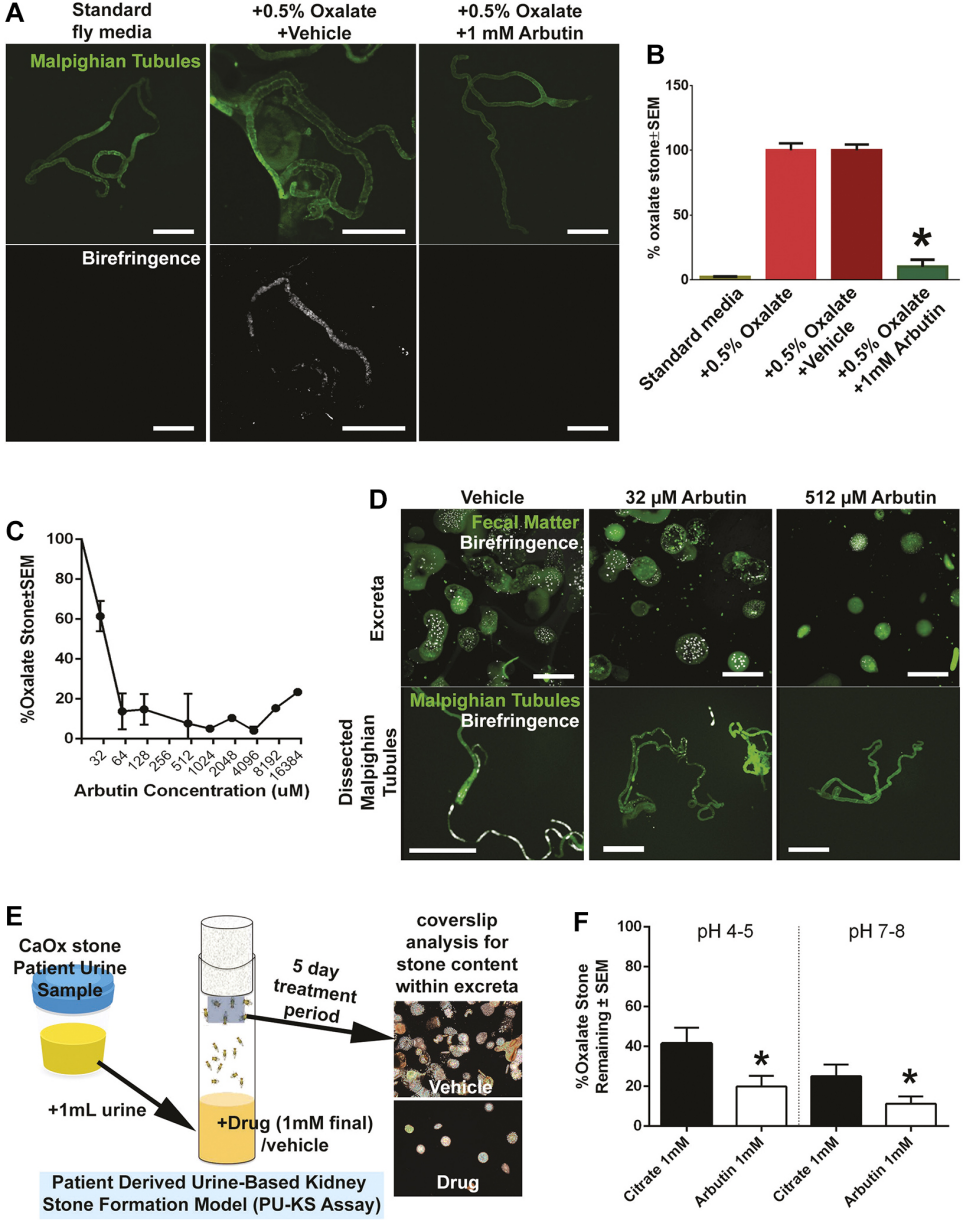
**Arbutin and its antilithogenic effects on oxalate-based calculi.** (A) Polarized microscopy of MTs treated with arbutin compared with standard medium and oxalate-supplemented fly medium. (B) Percentage of fecal excreta area that contains birefringence signal/calculi when flies are grown in various fly medium treatments, including arbutin. *P<0.01 with two-way ANOVA, Scheffe α correction. (C) Dose-dependent inhibition of oxalate-based fecal excreta deposited by *Drosophila* with various concentrations of arbutin. (D) Microscopy of coverslips deposited with calculi-rich fecal excreta and dissected MTs when fly medium is supplemented with 0, 32 and 512 µM arbutin. (E) Schematic of patient urine-based kidney stone *in vivo* formation assay. Patient urine samples are added to fly media with and no other supplementation. Coverslips containing calculi-rich fecal excreta are analyzed via microscopy. Vehicle image for coverslip analysis is DMSO sample reproduced from Fig. 3B; drug image is from the image labeled 2 in Fig. 3B. (F) The patient urine-based kidney stone *in vivo* formation assay was used to compare potassium citrate with arbutin at various pHs. Coverslips containing calculi-containing fecal excreta were analyzed by birefringence microscopy. *P<0.05, two-way ANOVA, Scheffe α correction.

Arbutin is a glycoside with a hydroxyquinone side chain with no previous reports regarding interactions with oxalate or calcium. When supplemented to fly medium+0.05% sodium oxalate, arbutin at 1 mM resulted in a near abrogration of calculi deposits in dissected MTs when compared with controls (fly medium+0.5% oxalate±DMSO) (Fig. 4A,B). Calculi content deposited in fecal excreta significantly decreased at >64 µM arbutin (Fig. 4C) with a calculated half-maximal inhibitory concentration (IC_50_) of 40 µM. Observed decreases in calculi content in fecal excreta corresponded with decreases in calculi content in MTs (Fig. 4D, 32 µM and 512 µM arbutin treatment).

In addition, urine samples from human patients with recurrent calcium oxalate kidney stones were added to fly medium with no sodium oxalate supplementation (Fig. 4E). *Drosophila* incubated in this manner generated calculi in the same manner as those incubated with fly food supplemented with sodium oxalate. This patient-urine-based model (*n*=10 patients) was used to compare the antilithogenic effect of arbutin compared with that of potassium citrate, an established therapy for oxalate-based kidney stone patients. Using 1 mM final concentrations in fly medium, arbutin administration led to a significant decrease in stone burden via fecal excreta assay in comparison with citrate, regardless of pH. Various pHs were evaluated in order to recapitulate the pH of human urine in the kidney and bladder (pH 4-6), with a greater reduction in calculi formation observed at pH 7-8.

### Arbutin binds to both free calcium ions and oxalate

**Fig. 5.**
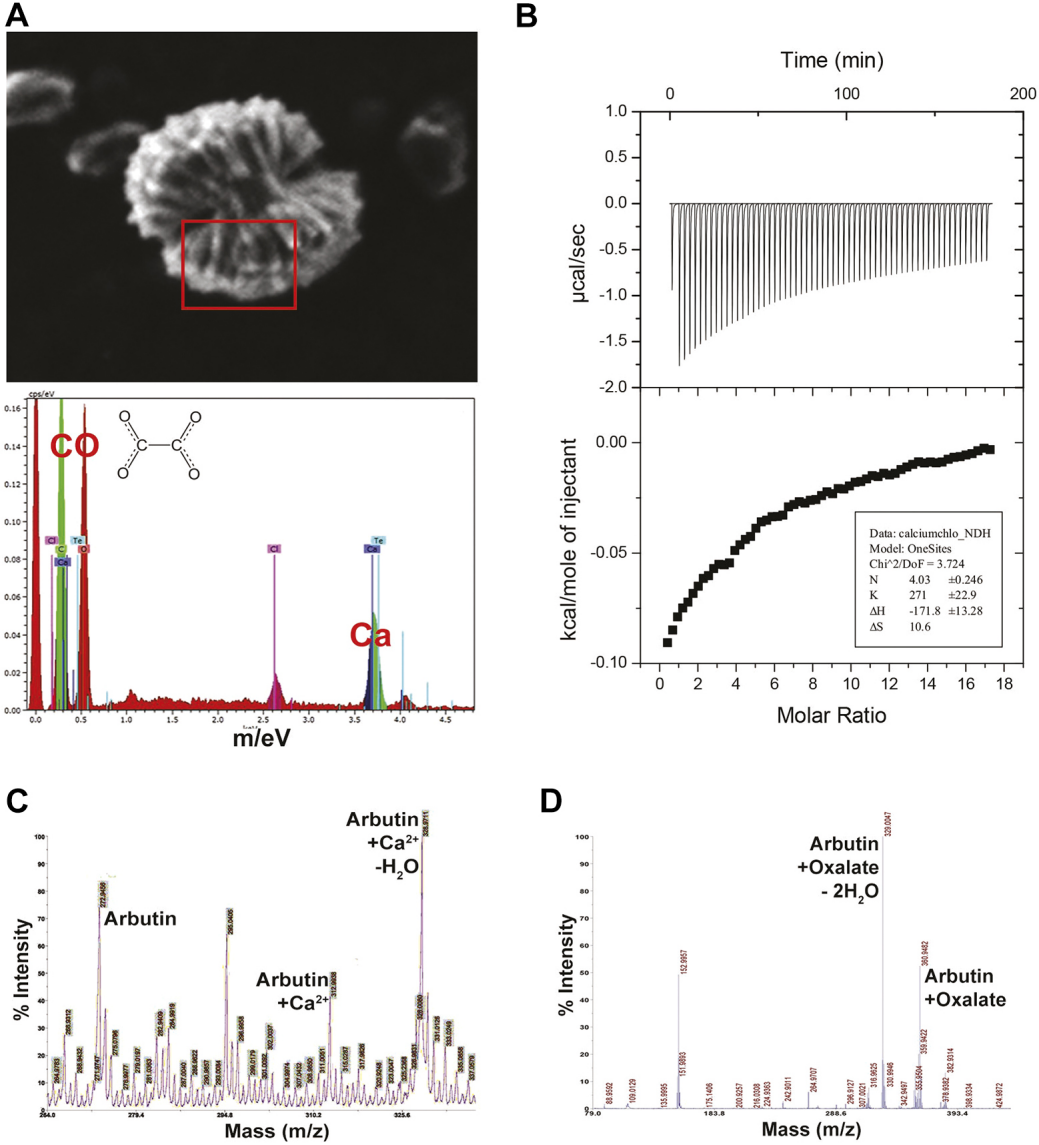
**Arbutin and its interactions with calcium and oxalate.** (A) SEM image of arbutin complexed with calcium. EDX spectra reveal four calcium ions for every molecule of oxalate. (B) Isothermal titration calorimetric analysis of arbutin and calcium chloride, revealing a molar ratio of four calcium ions for each arbutin molecule. (C) Matrix-assisted laser desorption/ionization (MALDI) spectrum of calcium and arbutin complexes formed in solution. (D) MALDI spectrum of arbutin and oxalate complexes formed in solution.

Arbutin was incubated with calcium chloride and then submitted to scanning electron microscopy (SEM), revealing clusters of arbutin and calcium (Fig. 5A, top) in a 1:4 stoichiometry, as determined by EDX spectroscopy (Fig. 5A, bottom). Isothermal calorimetry was used to determine whether a specific chemical interaction occurs between two agents of interest, with the release of heat indicative of chemical bonding (exothermic). Titration of either arbutin or calcium chloride to the other component revealed an exothermic reaction that plateaued at a molar ratio of 4.03 (Fig. 5B). High-performance liquid chromatography (HPLC) and mass spectrometry of arbutin and calcium chloride mixtures revealed peaks representing free arbutin, arbutin complexed with calcium ion, and arbutin complexed with calcium ion and water (Fig. 5C). HPLC and mass spectrometry of arbutin and sodium oxalate also revealed peaks representing arbutin+oxalate+water and arbutin+oxalate.

### Arbutin binds to the surface of oxalate-based nanocrystals

**Fig. 6.**
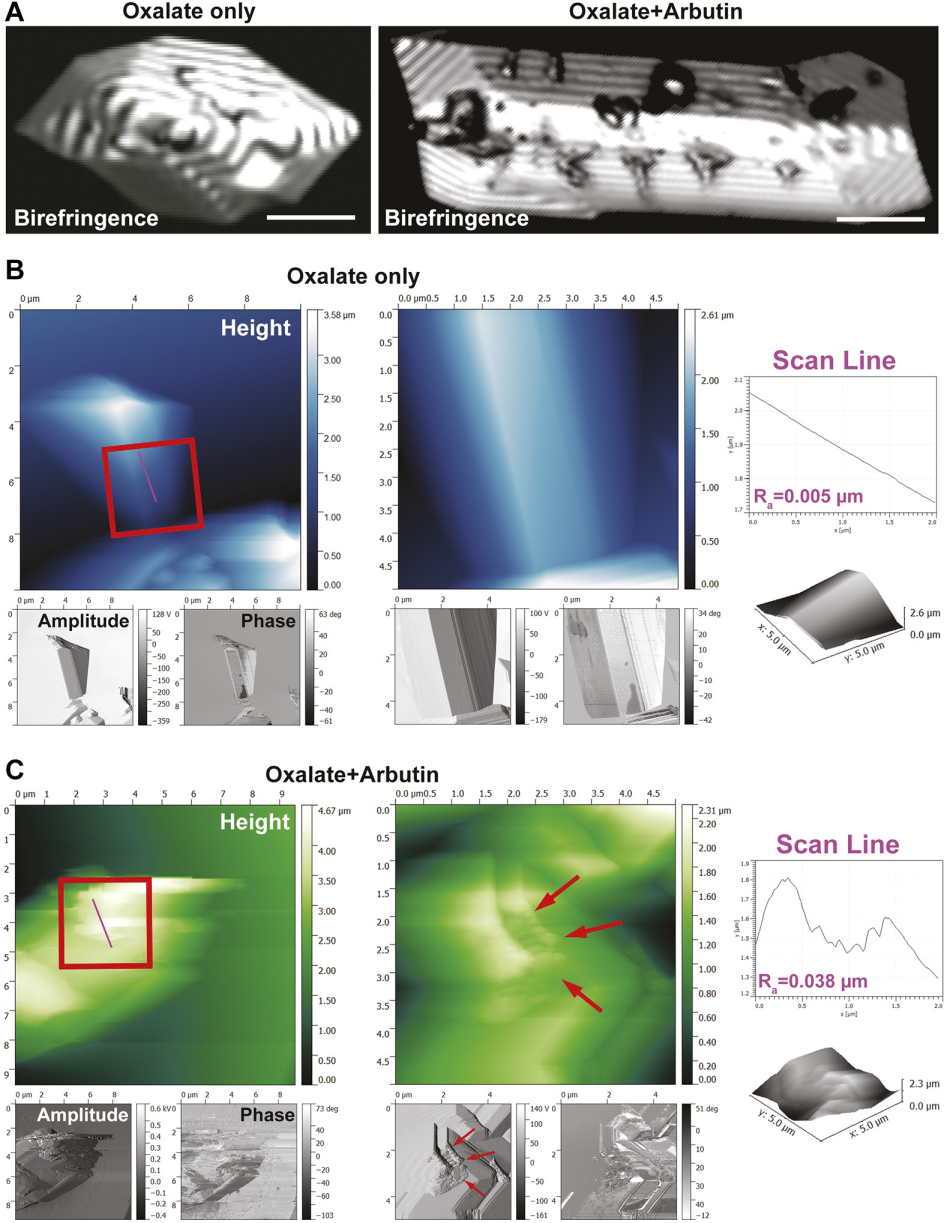
**Calcium oxalate and arbutin crystal structure interaction analysis.** (A) Confocal birefringence images of pure oxalate crystals prior to arbutin exposure (left), and following exposure to arbutin (right). Scale bars: 10 µm. (B) Atomic force microscopy (AFM) image of pure oxalate crystals. Insets in the middle panel provide higher magnification views of the crystal surface. Scan line analysis of the height channel within the inset reveals a smooth topography. (C) Oxalate crystals exposed to arbutin reveal a highly active surface topography decorated with arbutin drug molecules (red arrows). Insets in the left panel reveal a rough surface topography, as shown by scan line analysis of the height channel.

Large sodium oxalate crystals were formed for confocal birefringence microscopy to determine whether arbutin binds directly to the surface of calculi. Sodium oxalate calculi imaged in this manner revealed polygonal and smooth surfaces on oxalate-based crystals (Fig. 6A, left). Incubation with arbutin resulted in crystal surface aberrations (Fig. 6A, right). To confirm that arbutin binds to the surface of oxalate-based crystals, atomic force microscopy (AFM) was performed on both crystal types, with oxalate-only crystals revealing a smooth and uninterrupted surface according to scan line roughness analysis (Fig. 6B). Oxalate crystals treated with arbutin revealed a highly active crystal topography (Fig. 6C, red arrows), with a roughness index higher than in the untreated crystal. Oxalate-based crystals were also significantly smaller than nontreated crystals, based on AFM volumetric analysis (Fig. 6B,C).

### Arbutin inhibits oxalate-based toxicity *in vitro*

**Fig. 7.**
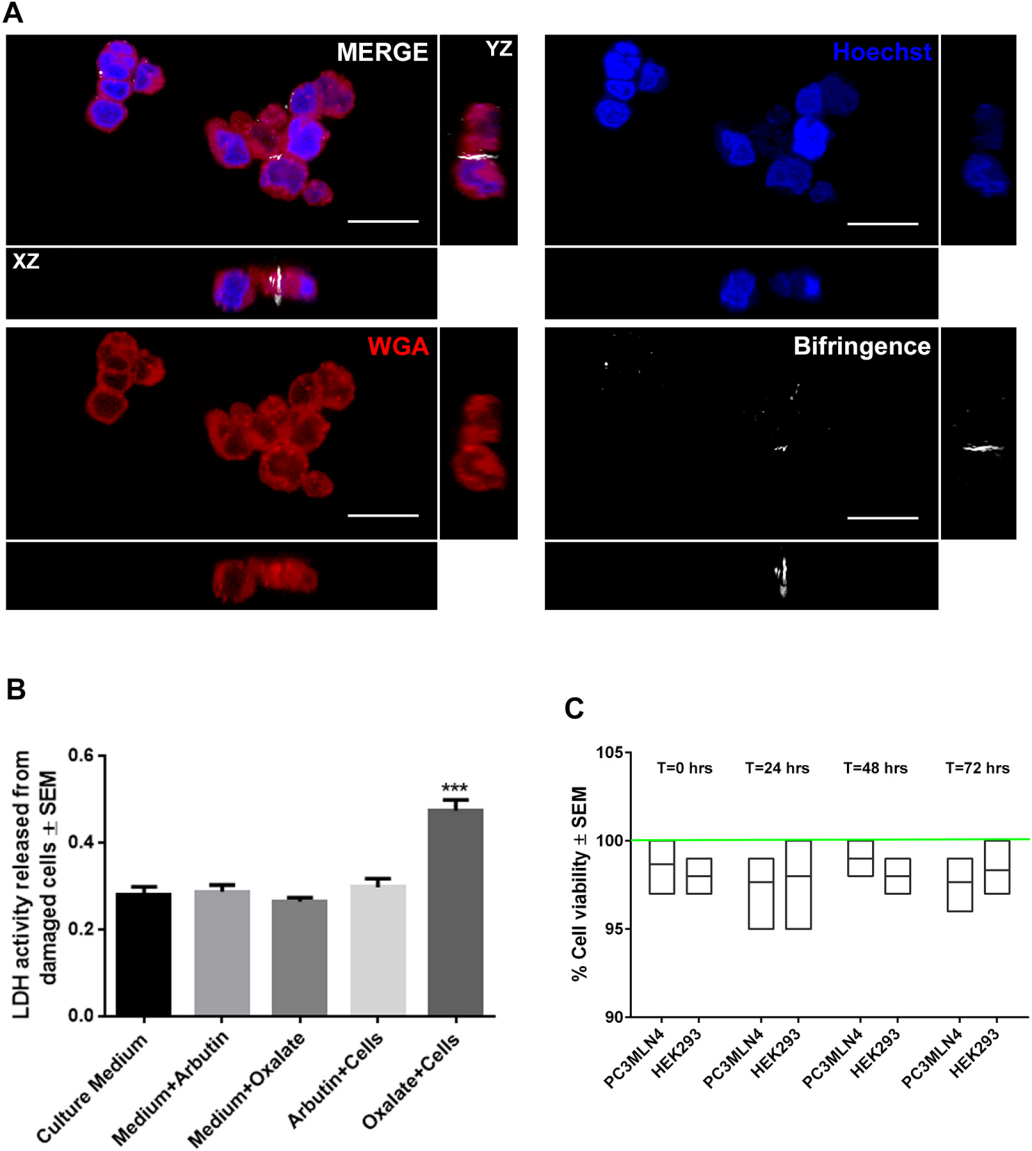
**Cytotoxicity of arbutin and oxalate on human kidney epithelial cells.** (A) Confocal fluorescence images of HEK293 cells stained with CellTracker Red to label the cell surface and Hoechst to label the nuclei. HEK293 cells were treated with 20 µM sodium oxalate for 30 min. Birefringence signal was observed within HEK293 cells (white signal). Scale bars: 25 µm. (B) Activity of LDH released from HEK293 cells after 30 min incubation with oxalate in the presence or absence of arbutin. (C) Cell viability in arbutin-treated HEK293 and PC3MLN4 cells over a 3-day period. Green line represents control normalized to 100% at that same timepoint. ***P<0.05, two-way ANOVA, Scheffe α correction.

Human embryonic kidney epithelial (HEK) cells grown *in vitro* incubated in 20 μM sodium oxalate were associated with significantly lower cell viability rates than those grown in control conditions, with some nanocrystals observed within cells over the incubation period (Fig. 7A). However, addition of arbutin (1 mM final) into the medium ameliorated this effect, with no impact on cell viability or lactate dehydrogenase (LDH) activity compared with arbutin treatment alone (Fig. 7B,C). No birefringent signal was observed within these cells.

## DISCUSSION

Aside from the divalent ions, calcium and magnesium, there are currently no known sequestrants for free and bound oxalate, the principal component of calcium oxalate calculi. Despite the limitations of *D. melanogaster* as a pre-clinical model of nephrolithiasis, we hypothesized that it could have some value as a high-throughput screening platform for identifying small molecules that inhibited calcium oxalate calculus formation *in vivo*, owing to the predominantly acellular metabolic and biochemical nature of nephrolithiasis. To our knowledge, this is the first high-throughput chemical library screen using *D. melanogaster*. This screening platform relies on the dietary administration of candidate drugs to observe changes in calculus composition via the described fecal excreta assay. This innovation permitted an unbiased ‘drop out’ screen to be performed for each candidate drug when ingested. Another key innovation was the use of a natural compound library that focused the screen to compounds that would not elicit a nephrotoxic effect, which is a current drawback of standard therapies of calculi. Screening a library of 360 different small molecules led to the identification of two compounds of antilithogenic activity, with arbutin producing the greatest effect. These antilithogenic effects were in magnitude to those observed with citrate treatment, further supporting the candidate ‘hits’.

The percentage of candidate hits that resulted in significant toxicity and decrease in *D. melanogaster* viability with the chosen screening dose was unexpected. This meant that ≥7.8% of screened compounds should have been evaluated at a log-fold lower dose, meaning that a proportion of these could have been drug candidates that otherwise were as effective as arbutin but at a lower dose. Re-screening these was not performed. This is a flaw in the study design because it would have prompted more screens of the library with no true termination point. The dose used was similar to other screening doses and yielded an acceptable viability rate of 92%. Upon completion of the first pass of the library screen, the set of candidate drugs discovered led to focused studies on a single compound. Discovery of an antilithogenic compound validated the capability of the screen, which was the main goal of the study.

Arbutin is currently used as a nonpharmaceutical herbal supplement in the form of a plant-based extract [Bearberry Leaf Dry Extract (BDLE)] for the prevention of urinary tract infections (Schindler et al., 2002). It is also an active ingredient in skin lightening creams, although a risk-benefit approach has been advised with use in this role owing to a skin sensitization effect (O'Donoghue, 2006). It is also present in significant quantities in wheat-based products, pears, coffee and tea. Interestingly, recent studies have associated the regular consumption of coffee and certain teas with a lower risk of stone formation (Ferraro et al., 2014; Stamatelou et al., 2003). A higher urinary excretion rate of calcium, decreased calcium oxalate supersaturation and decreased urinary oxalate was seen in caffeinated coffee drinkers (Ferraro et al., 2013, 2014).

The pharmacokinetic profile of arbutin is partially established; it is reported to be heavily absorbed from the gastrointestinal tract and is bioavailable as HQ by-products [Chemical Information Review Document for Arbutin (CAS No. 497-76-7) and Extracts from Arctostaphylos uva-ursi; https://ntp.niehs.nih.gov/ntp/htdocs/chem_background/exsumpdf/arbutin_508.pdf]. In previous human studies, 4 h after an oral dose of arbutin, ∼70% of the administered arbutin dose was retrieved in the urine as HQ conjugates, demonstrating renal excretion (Glöckl et al., 2001). The remainder could potentially be accounted for as free arbutin, available to interact with formed calcium oxalate and free subcomponents in the urine. No data on the plasma and urine bioavailability of free arbutin are presently available, possibly due to detection limitations of HPLC-based assays utilized in prior studies.

Arbutin has been described to readily degrade into the split components D-glucose and HQ (and conjugates) in acidic environments by Deisinger et al. (1996). An enteric coated tablet vehicle could thus potentially be utilized to bypass hydrolysis by stomach acids and improve oral bioavailability. However, our preliminary nuclear magnetic resonance spectroscopy (NMR) studies of arbutin in acidic conditions suggest that arbutin is stable and able to bind to oxalate whilst in solution, despite the low pH (data not shown). From available toxicology data, arbutin has been shown to have a low risk of acute and chronic toxicity by oral dosing in rat- and mouse-based experiments with no remarkable effects observed. Although no genotoxic effects were reported in *in vitro* or *in vivo* studies (Scientific Committee on Consumer Products: Opinion on β-Arbutin; https://ec.europa.eu/health/ph_risk/committees/04_sccp/docs/sccp_o_134.pdf), further studies are needed to complete the toxicological and pharmacokinetic profile of arbutin and HQ.

The mechanism of arbutin's antilithogenic action was evaluated *in vitro* on calcium oxalate crystals, calculi formed by *D. melanogaster*, and calculi induced in *D. melanogaster* by dietary supplementation of patient urine content. The mechanism of action appears to be twofold: arbutin binds to four calcium ions and it can bind to oxalate in a 1:1 stoichiometric relationship. The ability of arbutin to interact directly with oxalate is novel; only divalent ions such as calcium have previously been shown to interact with oxalate. Experiments with fly medium supplemented with sodium oxalate (0.5%) revealed antilithogenic abilities at concentrations as low as 64 µM, suggesting that it directly binds to oxalate, as confirmed by mass spectrometry. Arbutin also binds directly to pre-formed oxalate-based calculi, as determined by AFM and mass spectrometry, disrupting crystal lattice structure. Therefore, it appears that arbutin's antilithogenic activity is due to its binding to key components of calculi: divalent ions (calcium and magnesium) and oxalate. Translationally, the mode of action of arbutin could be binding to soluble oxalate in the gastrointestinal tract or in the blood. It is possible that arbutin's antilithogenic effect could be via reduction of dietary oxalate and, in the presence of a pre-formed stone, complex formation on the stone surface to inhibit further crystallization.

The observed effects of arbutin in these experiments raise intriguing questions about the potential for this agent to be used as a strategy to prevent and/or treat pre-existing human calcium oxalate stone disease. In patients at risk for calcium oxalate stone formation, arbutin could, through competitive inhibition, prevent calcium oxalate binding.

The fecal excreta calculi assay performed via the *D. melanogaster* model of human nephrolithiasis is a highly promising *in vivo* screening platform for the identification of antilithogenic compounds. It is flexible and scalable to screening of thousands of candidate compounds at minimal cost. The use of birefringent signals to quantitate stone burden via fecal excreta deposited onto coverslips is an innovation that is cost effective and does not require any additional processing or staining. It also entails that other birefringent calculi, such as uric acid calculi, are also amenable to this type of chemical library screen. This screening platform provides several avenues of opportunity to narrow down large numbers of potentially overlooked candidate compounds with possible clinical applications for further evaluation.

## MATERIALS AND METHODS

### *Drosophila* stocks

All fly stocks were reared on standard medium consisting of agar, yeast, milk powder, corn meal and corn syrup, at 25°C with a 12-12 h light-dark cycle at ∼40% humidity. Wild-type Canton S [Bloomington #1, Bloomington *Drosophila* Stock Center (BDSC)] fly line was used for all experiments. For fluorescent tubule imaging, the responder lines UAS-RFP (Bloomington #32218, BDSC) driven by the GAL4 lines c42 (provided by J. Dow, University of Glasgow, Glasgow, UK) and URO-GAL4 (Bloomington #44416, BDSC) were utilized.

### Stone and urine sample collection

All experiments and sample collection procedures were approved by local hospital/institution research ethics board (REB) panels, and assigned as REB #105604. Patient-derived stone and urine samples were collected from consenting patients who underwent percutaneous nephrolithotomy. Approximately 10 ml of the urine was collected via cystoscopy and subsequently stored at −80°C. Stone samples were collected using either duckbill forceps or an endoscopic grasping basket and deposited directly into a sterile container and stored at −20°C. Patient data, stone and urine analysis data, including oxalate crystal data, were previously presented in an independent report (Gavin et al., 2016).

### Synthesis of alendronate-FITC and its negative isotype control notdronate-FITC

Bisphosphonates are well known to bind tightly to the surface of bone. Several groups have conjugated bisphosphates to a fluorophore, which has been used to image the localization of these bisphosphate groups to bone (Gavin et al., 2016; Cole et al., 2014). Our imaging agent consists of the commercially available bisphosphonate drug alendronate conjugated to the fluorescent dye FITC. As a negative control, the bisphosphonate group of alendronate was removed, leaving 4-amino-1-butanol conjugated to the dye. The amine [alendronate or 4-amino-1-butanol (Alfa Aesar)] was dissolved in saturated NaHCO_3_(aq). The NHS-ester of fluorescein (Thermo Fisher Scientific) was dissolved in dimethylformamide (Fisher BioReagents) and added to the amine. The reaction was stirred in the dark overnight. The solution was purified by reverse-phase flash column chromatography (Biotage Isolera One, 12 g C18 SNAP, methanol in water 0-100%) and the product-containing fractions were lyophilized. The lyophilized powder was dissolved in water (Milli-Q, 18.2 MΩ cm), dialyzed with water overnight (Float-A-Lyzer G2, Spectrum Labs), and lyophilized to yield the fluorescent dye conjugates. All reagents were provided in powder form and stored in a −20°C freezer in an opaque container.

### Testing specificity of fluorescent probes to hydroxyapatite and calcium-based stones

Synthetic hydroxyapatite particles (Sigma-Aldrich) and pulverized samples of infrared spectroscopy-confirmed human calcium oxalate stones were stained. Then, 100 mg of sample was incubated in an Eppendorf tube with 200 ml 0.1 mM alendronate-FITC, 0.1 mM notdronate-FITC and PBS as a control. Afterwards, the samples were centrifuged (Eppendorf) at 14,000 ***g*** for 5 min, the supernatant was removed and three subsequent washes were performed with distilled water to remove any unbound dye. The samples were then mounted onto glass slides with coverslips for confocal microscopy.

### Diet-induced calcium oxalate stone formation

Stone-forming diets were prepared using the lithogenic agents sodium oxalate (Sigma-Aldrich) and ethylene glycol (Sigma-Aldrich). These compounds were added to the water during the preparation of fly food medium. Four concentrations were prepared for each lithogenic agent at 0.05%, 0.1%, 0.5% and 1% w/v ratio and stored at 4°C until required. Twenty newly eclosed flies were added to 15 narrow vials containing either of the lithogenic diets or standard food medium as a control. The flies were fed for 7-14 days, after which they were removed for staining and imaging.

### Generation of fluorescent MTs

Fluorescent MTs were generating by crossing fly lines expressing UAS-RFP with the driver lines c42 and URO-GAL4. URO-GAL4 drives expression in the principal cells of the MTs and line c724 in the stellate cells. Age-matched newly eclosed virgin females carrying UAS-RFP were crossed with newly eclosed males from the driver lines. After 2-3 days of mating and the laying of eggs, the flies were discarded and the transgenic variants allowed to mature. After maturation, these flies were reared on either the lithogenic diets or the standard control diet for 14 days. They were subsequently dissected and processed for imaging.

### *Ex vivo* staining and imaging of *Drosophila* MTs

Flies were euthanized by carbon dioxide narcotization using the Flow Buddy CO_2_ System (Flystuff). The MTs were carefully dissected under a stereomicroscope in a Sylgard (Dow Corning)-lined Petri dish using Schneider's media (Sigma-Aldrich). The tubules were mounted on a fresh Petri dish and minutien pins (Fine Science Tools) were used to anchor the ends. Each tubule was incubated in either 200 ml 0.1 mM alendronate-FITC, 0.1 mM notdronate-FITC or PBS for 30 min. Consequently, tubules were washed several times with PBS and then mounted for wide-field fluorescence imaging (Nikon), birefringence microscopy (Nikon), or resonance confocal microscopy (Nikon).

For the MTs expressing RFP, they were first stained with 4′,6-diamidino-2-phenylindole (DAPI) followed by our fluorescent probes alendronate-FITC and notdronate-FITC. The tubules were first imaged with normal light followed by polarized birefringent microscopy and finally imaged with a scanning laser with a confocal microscope. We used a Nikon TE2000 Inverted Microscope and a Nikon Confocal Microscope for imaging.

### X-ray diffraction spectroscopy

Approximately 150 flies fed lithogenic medium were dissected, their MTs extracted and stored in 1 ml PBS in an Eppendorf tube. Collected MTs were sonicated for 30 min in a solution consisting of proteinase K and 0.1% Triton X followed by centrifugation at 14,000 ***g*** for 10 min. The supernatant was discarded. The solids were washed thoroughly with double distilled water and re-centrifuged. Then, 2 µl sample was mounted on silica wafer for SEM and X-ray diffraction spectroscopy analysis.

### Lifespan study

*Drosophila* eggs were collected on grape juice agar plates in egg collection cages (Diamed Inc.). The eggs were washed multiple times with PBS and 32 ml egg solution was pipetted and deposited in vials containing standard medium. After 10 days, newly eclosed flies were anesthetized using carbon dioxide and separated by sex. Twenty flies of the same gender were added to ten vials containing varying concentrations of lithogenic agent or standard medium as a control. Flies were transferred to vials containing fresh food on alternate days and the number of deaths recorded. This was performed over a period of 60 days. Data were analyzed using the Statistical Package for Social Sciences for Mac (SPSS Version 21 Mac) and graphical data generated using Graph Pad Prism Software (GraphPad Prism 6).

### Quantification of calcium oxalate calculi via *D. melanogaster* excreta birefringence assay

Twenty newly eclosed flies were housed in 15-ml narrow fly vials with stone-forming medium containing 0.5% (w/v) sodium oxalate. A glass coverslip (18×18 mm) was suspended from the cotton tube plug to allow for the collection of fecal matter. Following a 14-day incubation period, the coverslip was extracted from each tube and each coverslip was directly imaged by confocal microscopy under polarized light. Control experiments were performed in a similar fashion with standard medium. The percentage area of birefringence for each coverslip was individually analyzed and quantified using NIH ImageJ software (Particle Analysis function).

### Drug library screening

Stone-forming medium containing 0.5% (w/v) sodium oxalate was prepared. Candidate compounds from our drug library (Lee et al., 2015; Rota et al., 2017; Spagnuolo et al., 2010) were added to 10 ml of the medium up to a total concentration of 20 µM drug. This drug library consisted of 360 different compounds diluted to 1 mM in DMSO and stored at −80°C. Vehicle controls such as PBS and DMSO were used and randomly inserted into the library. Five milliliters of the total 10 ml medium were decanted into two separate 15 ml narrow vials to perform further experiments as duplicates. Twenty newly eclosed flies were added to each vial. Following a 14-day incubation period, each coverslip was removed and mounted onto glass sides in preparation for imaging. Each slide was directly subjected to confocal microscopy under polarized light. The percentage area of birefringence was quantified and compared with birefringence data from control experiments with separately prepared 0.5% sodium oxalate media with no addition of candidate drugs.

### Dose-response relationship of hydroquinone arbutin and calcium oxalate stone formation

Thirty milliliters of 0.5% (w/v) sodium oxalate medium was prepared with 19.3 mM arbutin. A tenfold serial dilution was performed to obtain 15 ml medium per dilution. Each dilution was divided into three narrow fly vials to yield triplicates. Twenty newly eclosed flies were added to each vial with a glass coverslip (18×18 mm) suspended from the cotton vial plug to collect fecal matter. After a 14-day incubation period, each coverslip was extracted and stone burden was quantified as previously described to observe the dose-response relationship between arbutin concentration and percentage area of birefringence.

### Patient-derived urine-based calcium oxalate stone induction in *D. melanogaster*

Urine samples were collected from recurrent calcium oxalate stone-forming patients and centrifuged at 1000 ***g***. One-milliliter aliquots of supernatant were prepared and stored at −80°C for further analysis. Ten milliliters of standard medium were prepared and thoroughly mixed with 1 ml patient urine; this was separated into two individual narrow vials (5 ml per vial). Twenty newly eclosed flies were added to each vial. A glass coverslip (18×18 mm) was suspended from the cotton vial plug to collect fecal matter. This experiment was repeated with patient urine-containing diet in the presence of 3 mM arbutin. Following a 14-day incubation period, each coverslip was extracted and imaged as described previously. Stone burden on each coverslip was quantified via the aforementioned birefringence assay and results were expressed as mean±s.e.m. Percentage stone burden was analyzed using one-way analysis of variance (ANOVA) (Prism 6, GraphPad Software, San Diego, CA, USA).

### *In vitro* patient-urine based analysis of arbutin and calcium oxalate interactions

Fifty microliters of patient urine were combined with 45 µl 20 mM HEPES buffer (pH 7-8). Five microliters of 20 mM arbutin were added to each solution to form a 1 mM final concentration of the drug. Immediately after addition of the drug, 5 µl of each sample was spotted on a glass slide and birefringence data were imaged on confocal microscope. Each sample was incubated at 37°C for 24 h. Five microliters of the sample were identically collected and reimaged. Data on percentage area of birefringence were analyzed as previously described. This experiment was repeated in sodium acetate/acetic acid buffer at a pH of 4 to observe the efficacy of arbutin in acidic environments. Reduction of percentage stone reduction was analyzed using one-way ANOVA. Control experiments were conducted using Quick Fix Plus synthetic urine from Spectrum Labs, Cincinnati, OH, USA.

### Isothermal calorimetric titration of arbutin and calcium chloride

To investigate the interaction between arbutin and calcium ions, 75 mM calcium chloride and 1 mM arbutin solutions were prepared in 20 mM HEPES buffer at pH 7.4 in preparation for isothermal titration calorimetry. The isothermal calorimetric titration (ITC) binding experiments were performed in the Nano ITC Standard Volume with a fixed gold cell by titrating 250 µl injections of 75 mM calcium chloride solution into 1 mM arbutin solution at 350 s intervals for 1800 s (30 min) with a stirring speed of 300 rpm. The user-adjustable auto equilibrate function was used to ensure a stable baseline prior to injection of titrant. ITC data were analyzed with NanoAnalyze Software (TA Instruments).

### Matrix-assisted laser desorption/ionization (MALDI) of arbutin-calcium and chloride arbutin-sodium oxalate reaction mixtures

To further investigate the interactions of arbutin with calcium and oxalate ions, two separate reaction mixtures containing 1 mM arbutin and 75 mM calcium chloride solution and 1 mM arbutin: 75 mM sodium oxalate in 20 mM HEPES buffer (pH 7.4) were prepared. These reaction mixtures were incubated at 37°C for 24 h and submitted for mass spectrometry at the Mass Spectrometry Facility, University of Western Ontario, London, ON, Canada. Samples were analyzed on a Bruker Daltonics Reflex IV research grade MALDI time-of-flight (TOF) instrument equipped with a linear/reflectron mass analyzer and postsource decay (PSD).

### Crystallization of sodium oxalate in the presence and absence of arbutin

To determine the effect of arbutin on the crystallization of calcium oxalate, crystals of calcium oxalate were prepared by vapor diffusion, and 10 mM sodium oxalate and calcium chloride solutions were prepared in ultrapure water in separate glass vials of different sizes. The smaller glass vial containing the calcium chloride solution was placed inside the larger vial containing sodium oxalate. The system was loosely covered and allowed to rest for a 3-week period with minimal disturbance. This experiment was repeated in the presence of 1 mM arbutin. Crystals formed in the outer oxalate-containing vial were collected to for further processing via confocal microscopy and AFM.

### Cytotoxicity assay of sodium oxalate and arbutin on human kidney epithelial cells

HEK cells (HEK293; ATCC^®^ CRL-1573™), were cultured in a six-well plate containing 18-mm diameter glass coverslips to 50% confluence. Following aspiration of culture medium, the cells were incubated in a chloride-free solution containing 130 mM potassium gluconate, 5 mM glucose, 20 mM HEPES (pH 7.4) and 20 μM sodium oxalate for 30 min. The treated medium was then removed and stored at 4°C for further analysis. Cells were washed with Cl^−^-free buffer and stained with 20 µM CellTracker Red CMTPX Dye (Thermo Fisher Scientific) for 15 min. Cells were triple washed with Cl^−^-free buffer and coverslips were mounted with ProLong^®^ Gold Antifade Mountant with DAPI (Thermo Fisher Scientific), without permeabilizing, in preparation for confocal microscopy. Samples were imaged using a Nikon A1R+ confocal microscope (1.2 au) with a 20× dry or 60× oil immersion lens and presented using NIS Elements software (Nikon).

The cytotoxicity of HEK293 cells in sodium oxalate medium with or without arbutin was determined by quantifying lactose dehydrogenase content liberated by damaged cells using a Cytotoxicity Detection KitPLUS (LDH) (Roche), following the manufacturer’s instructions. Absorbance readings were taken at 492 nm using a BioTek PowerWave HT Microplate Spectrophotometer.

## Supplementary Material

Supplementary information

First Person interview
