# Supplementary material for: Drosophila melanogaster as a function-based high-throughput screening model for antinephrolithiasis agents in kidney stone patients
Source: Dis Model Mech. 2018 Nov 16;11(11):dmm035873. doi: 10.1242/dmm.035873 (PMC6262805; doi:10.1242/dmm.035873)
Supplement: Supplementary information [file dmm-11-035873-s1.pdf]

FIRST PERSON

First person – Sohrab Ali and Thamara Dayarathna

First Person is a series of interviews with the first authors of a selection of papers published in Disease Models & Mechanisms, helping early-career researchers promote themselves alongside their papers. Sohrab Ali and Thamara Dayarathna are co-first authors on '*Drosophila melanogaster* as a function-based high-throughput screening model for antinephrolithiasis agents in kidney stone patients', published in DMM. Sohrab and Thamara conducted the research described in this article while Sohrab was a graduate student and Thamara a postdoc in the lab of Dr Hon Leong at the University of Ottawa, Canada, investigating endourology and the pathogenesis of stone disease. Sohrab is now continuing his surgical training in urology at the University of Ottawa, Canada. Thamara is a Research Scientist in the lab of Dr Paul Spagnuolo at the University of Guelph, Canada, working towards translating bench work research to bedside applications by studying the mode of action of small and macromolecule drug candidates in disease control mechanisms.

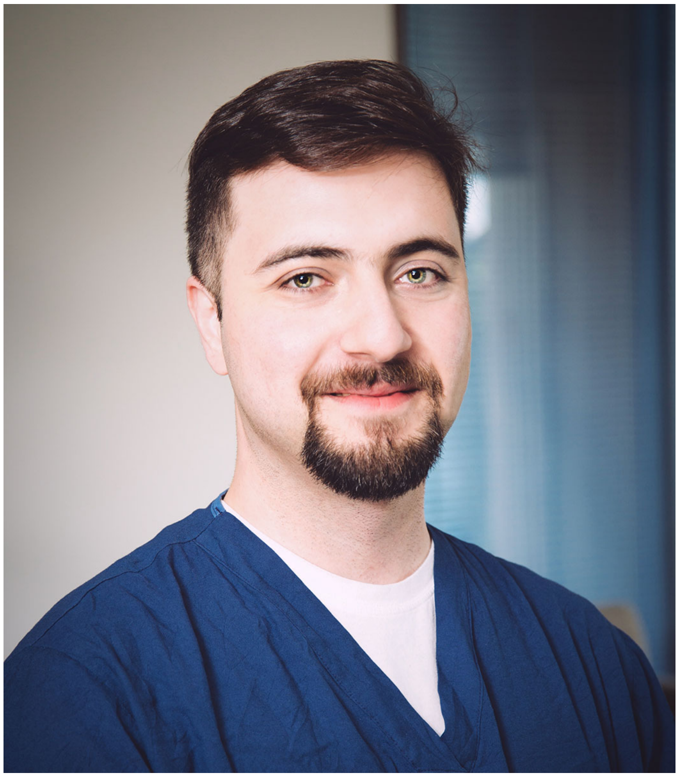

Sohrab Ali

How would you explain the main findings of your paper to non-scientific family and friends?

SA: Kidney stones are a common and often painful condition. Despite advances in the surgical treatment of kidney stones, very little research has been done on how and why these stones form in the first place. In this paper, we have used the common fruit fly as a novel model for researching kidney stones in order to find chemicals or compounds that might prevent the stones from forming. Although the fruit fly might seem like an unlikely candidate, they surprisingly have many things in common with humans. The fruit fly has two pairs of kidney tubules that are very similar to a human nephron, the single filtering unit in a human kidney. We fed the flies diets containing chemicals that caused them to form kidney stones. This allowed us to use microscopes and other novel imaging techniques to see these stones forming. We obtained a large library of experimental drugs that were used on the flies with kidney stones and discovered a compound, arbutin, that reduced stone formation. We believe that this research will provide a starting point for drug design and has the potential to impact the lives of patients with kidney stones in a very dramatic way.

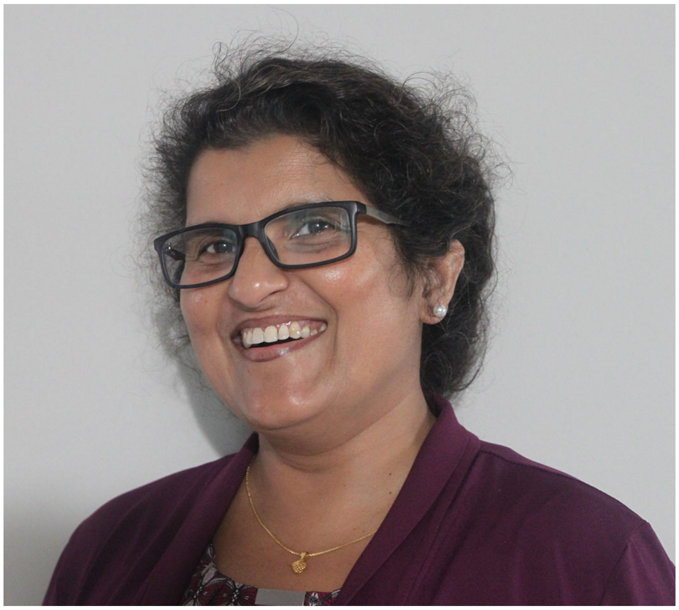

Thamara Dayarathna

Sohrab Ali's contact details: The Ottawa Hospital - General Campus, Division of Urology, 501 Smyth Road, Ottawa, ON K1H 8L6, Canada
Thamara Dayarathna's contact details: Department of Food Science, University of Guelph, 50 Stone Road East, Guelph, ON N1G 2W1, Canada.
E-mails: soali@toh.ca; thamara.dayarathna@sjhc.london.on.ca

TD: Kidney stone disease is a common, painful disorder of the urinary tract affecting more than 10% of the population in developed countries. The incompleteness of our overall understanding of the disease and a lack of proper drug treatment means that research in this field is limited. Recent advancements in living organism and animal-free research have led to the study of kidney stone-forming mechanisms, and novel drug candidates to treat the disease and prevent its recurrence. In this paper we used the common fruit fly model as the living system to screen potential candidates for kidney stone treatment, and animal-free systems to study the mechanism of action of these drugs on stones. Under these circumstances we monitored changes in the chemical composition and physical appearance of kidney stones in the presence of the main drug candidate, arbutin, to test the drug efficacy on stone formation. The fruit fly model allows us to study the process of kidney stone development quickly, as they have a very short life span, and their urinary tract has structural similarities to a single filtering unit of the

human kidney. We believe that the findings of this research provide a good starting point for designing drug candidates involved in mitigating kidney stone formation, with the potential to also develop powerful treatments for more serious renal disorders such as oxalosis. Hence, our findings have the potential to help millions of people around the world affected by kidney stones, changing the face of current practice.

What are the potential implications of these results for your field of research?

SA: Here, we showcase the utility of the emerging *Drosophila melanogaster* model for human nephrolithiasis. We have discovered an anti-lithogenic agent, arbutin, for calcium oxalate nephrolithiasis using novel imaging techniques and high-throughput drug screening. This work will serve as a basis for rational drug design and further translational research in this area.

TD: In this paper we focused on three main investigations: (1) the utility of the emerging *D. melanogaster* model for human nephrolithiasis, (2) the efficacy of the fly model in high-throughput drug screening and (3) the mode of action of drug candidates on oxalate stones. These research findings have initiated an arbutin-based drug design for oxalate stones and currently we are progressing towards translating this research to bench-side.

What are the main advantages and drawbacks of the model system you have used as it relates to the disease you are investigating?

"[T]he *Drosophila* community is a collegial community with researchers and investigators eager to reach out and share their work."

SA: *Drosophila melanogaster* is a versatile model organism with a rich history in translational research spanning more than a century. It is for this reason that it was an obvious choice for our research in kidney stone disease. The advantages include cheap and easy acquisition from the *Drosophila* stock centers in Indiana, USA and Kyoto, Japan. They are easy to work with and maintain at minimal expense. Their short life cycles allow for faster experiments and large-scale high-throughput screening. The *Drosophila* genome is fully mapped with publicly available databases and human orthologue prediction tools. Its genome is easily modifiable with innovative genetic tools such as the GAL4 system. The *Drosophila* Malpighian tubules share striking similarity to the human nephron, which allows for the creation of the metabolic milieu required for stone formation. Finally, the *Drosophila* community is a collegial community with researchers and investigators eager to reach out and share their work. The main drawbacks include the challenge of designing experiments with true translatability to the human pathogenesis of kidney stone disease. Not all stone types have yet been established using this model.

What has surprised you the most while conducting your research?

SA: The most surprising aspect was the vibrant community of *Drosophila* researchers throughout the world. Meetings such as the Annual *Drosophila* Research Conference, where this project was presented, provided feedback and opportunities for collaboration that were instrumental in its fruition.

TD: For me, it was the day we saw the effect of arbutin on kidney stones secreted via human urine, and the molecular level interaction of arbutin with sodium oxalate ions that surprised me the most. This is because it confirmed that the *D. melanogaster* model, which can be used in high-throughput drug screening for stones, is a good representation of the human urinary tract as well as for stone formation. Along with this excitement, the feedback provided by the worldwide *Drosophila* research community in regard to our findings was dynamic and rewarding.

Describe what you think is the most significant challenge impacting your research at this time and how will this be addressed over the next 10 years?

SA: The most significant challenge for nephrolithiasis-related research is establishing clear pathogenic pathways for this multifactorial disorder. A better understanding of this will allow for development of more targeted therapeutics. There is now a growing body of research with new and innovative models such as *D. melanogaster* being used to better understand how and why kidney stones form. With more funding opportunities for early-career scientists we believe significant advances will be made in the next ten years.

TD: The most significant challenge for drug discovery is having the right disease model and establishing the right pathogenic pathways for the disease. The use of innovative models such as *D. melanogaster*, in concert with sophisticated technologies and biochemical/biomedical proficiencies with collaborators, will help all of us make significant advancements in the field of drug discovery in the next 10 years.

"The most significant challenge for drug discovery is having the right disease model and establishing the right pathogenic pathways for the disease."

What's next for you?

SA: Completing residency training in urology. Developing *D. melanogaster* models for other types of nephrolithiasis such as uric acid and cystine.

TD: To develop the drug, arbutin, to treat patients with oxalate stones and reduce the disease recurrence rate. In the long term, I am looking forward to becoming a research investigator focused on drug-disease modes of action.

Reference

Ali, S. N., Dayarathna, T. K., Ali, A. N., Osumah, T., Ahmed, M., Cooper, T. T., Power, N. E., Zhang, D., Kim, D., Kim, R., St. Amant, A., Hou, J., Tailly, T., Yang, J., Luyt, L., Spagnuolo, P. A., Burton, J. P., Razvi, H. and Leong, H. S. (2018). *Drosophila melanogaster* as a function-based high-throughput screening model for antinephrolithiasis agents in kidney stone patients. *Dis. Model. Mech.* **11**, dmm035873.
